# Automated sample tracking and parameter adaption for scanning laser optical tomography

**DOI:** 10.1371/journal.pone.0318974

**Published:** 2025-03-19

**Authors:** Hannes Benecke, Firas Almadani, Johannes Heske, Tobias May, Ludger Overmeyer, Sonja Johannsmeier, Tammo Ripken

**Affiliations:** 1 Life Sciences Department, Laser Zentrum Hannover e.V, Hannover, Germany; 2 InSCREENeX GmbH, Braunschweig, Germany; 3 Institute of Transport and Automation Technology, An der Universität, Garbsen, Germany; Rutgers University Newark, UNITED STATES OF AMERICA

## Abstract

Non-destructive, three-dimensional imaging techniques are of great importance in medicine as well as in technical analysis. In this context, it is of particular importance to generate reliable and repeatable results of high quality. This can be aided by automation of manual processes. One of these imaging techniques, the Scanning Laser Optical Tomography, currently requires manual sample alignment by the user to achieve the highest possible image quality. This alignment demands skillful hand-eye coordination as well as experience on the part of the user, and thus often leads to inconsistent imaging results. To overcome this problem, this paper presents a technique for software-based automation of this challenge. The sample is not physically aligned, but digitally detected and tracked during the acquisition. Residual motion artifacts that interfere with tomographic reconstruction are corrected using a second automation algorithm. The combination of the two new algorithms significantly improves the quality of imaging and also increases the reliability and degree of automation of the system, making it accessible to a wide range of users.

## 1. Introduction

The non-destructive examination of three-dimensional (3D) samples through tomography is a topic of significant interest in various technical and biological fields. Both fields demand meticulous sample preparation and highly precise tomographic equipment. In the realm of biological samples, optical tomography offers distinct advantages over traditional x-ray-based systems. This is primarily due to the utilization of fluorescence, which enables the targeted investigation of specific structures within a sample. Various optical tomographic techniques, such as Optical Projection Tomography (OPT), Optical Coherence Tomography, and Light Sheet Fluorescence Microscopy, are available and are tailored to specific applications [1–3]. Each of these devices offers unique advantages for their respective applications, but also comes with its own considerations pertaining to optical properties and limitations.

One noteworthy variation of optical tomographs is the Scanning Laser Optical Tomograph (SLOT) [4]. This device utilizes a weakly focused laser beam, which is scanned across a rotating sample. Similarly to OPT, the transmitted light is acquired and captured as a two-dimensional transmission image. Through a rotation of the sample, a dataset is acquired, which can be reconstructed as a tomographic 3D-model. The modular design of SLOT makes it a broadly applicable device exploiting various contrast mechanisms. However, there are specific constrains regarding the movement of the sample during the acquisition. In this work, two major challenges are investigated. The first is the alignment of the sample within the acquisition space, which affects the quality of an individual acquisition, based on the user’s experience: To acquire the highest possible optical resolution, the specimen’s axis of rotation must be aligned to its spatial center. The sample however is embedded in a rigid polymer cylinder prior to imaging, resulting in the need to manually adjust the placement of the entire block to move the sample into the correct space. This complicates the repeatability of measurements. The second challenge in terms of movement is the jitter of a sample during its rotation. Irregular movements of the sample are caused by mechanical influences, such as the wear of the rotation component or extrinsic shocks. Especially for small specimen the resulting motion artifacts can lead to blur in the reconstructed tomographic images.

To overcome these two major challenges, we present two individual approaches which aim for the cost-free improvement of the existing setup. The approaches utilize computer vision and post-processing algorithms to improve the image quality. The first method eliminates the need for specimen alignment without any further hardware and simultaneously ensures the highest optical resolution. The second method corrects any residual jitter of the sample and is aimed to be an automated approach, applicable for a broad variety of SLOT samples.

## 2. Materials and methods

### 2.1. Scanning laser optical tomography

Scanning Laser Optical Tomography is a versatile tool for imaging biological [5–7] and technical [8,9] samples on a micro- to mesoscale. The technique is analogous to computer tomography, using a laser instead of x-rays. It was developed in 2010 at the Laser Zentrum Hannover e.V. as a derivative of OPT. Instead of using a diffuse light source for imaging, SLOT utilizes a weakly focused laser beam, which is scanned across a sample. Since its development, various extensions have been developed to enhance the range of contrast mechanism, such as absorption, fluorescence, scattering or higher harmonic generation [10]. For the acquisition of a tomographic dataset, first a sample is prepared, then imaged and finally computationally processed with tomographic algorithms.

To prepare a sample for SLOT imaging, it must be optically cleared and mechanically fixed. To combine both steps, the Curing Resin-Infiltrated Sample for Transparent Analysis (CRISTAL) protocol [11] was developed. Optical clearing is achieved with the solvent-based CRISTAL protocol, using benzyl alcohol, by converging the refractive index of the sample’s different components. In a second step the sample is embedded in an optical adhesive in a cylindrical shape which stabilizes the sample mechanically after the glue is cured. The refractive index of the adhesive is adapted to the resulting refractive index of the sample after clearing.

**Fig 1:**
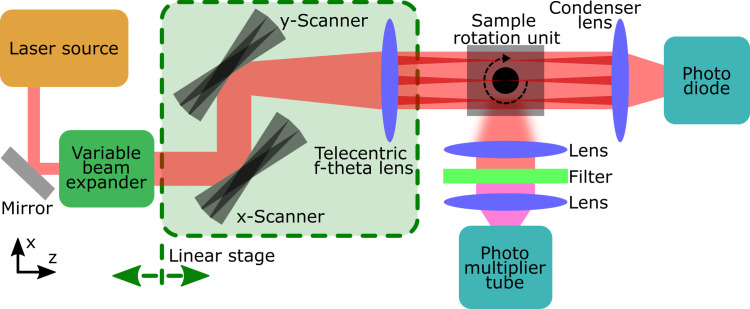
The basic Scanning Laser Optical Tomography (SLOT) setup [ 12]. A laser beam is adjusted in size, according to the imaging parameters. The beam is scanned and focused across a sample. The transmitted intensity of the laser is measured for distinct points across the sample, resulting in a transmission image of the sample. Fluorescence can be measured simultaneously. The sample is rotated in discrete angles around 360 degrees to acquire a dataset which can be reconstructed to a tomographic dataset.

After sample preparation, the SLOT acquisition can be started, see Fig 1. To do this, the output beam of a laser light source is first changed in its diameter and collimated with the aid of a variable beam expander. More details on this are explained in section 2.3. The adjusted beam is then directed towards the sample unit using an xy-scanner and a telecentric f-theta scanning lens. The lens ensures parallel beams and a flat focal plane. The previously cleared sample is placed into the sample unit at this point. The sample unit consists of a rotation stage, which rotates the sample 360° step by step during exposure, and a glass cuvette that encloses the sample. The glass cuvette is filled with a medium that has the same refractive index as the embedded sample. This allows the laser beam to be scanned two-dimensionally (2D) across the sample without being deflected in front of it, as each beam only encounters media with different refractive indices (namely air - glass - immersion medium) orthogonally. After the laser beam has interacted with the sample, the remaining intensity is measured using a photodiode. Scanning the sample thus produces a 2D transmission or absorption image. At the same time, any fluorescence emitted by the sample can be measured using a photomultiplier tube. For this purpose, the fluorescence light can be captured, filtered and recorded from any spatial direction. With this setup, a data set of 2D images of a rotating sample can finally be acquired. These are needed for the reconstruction into tomographic data sets in the next step.

The last step in the SLOT process chain is the reconstruction of the captured projection images using computer algorithms. Here, the data sets are first processed to ensure a high image quality that is as free of artefacts as possible [13]. The projections are then converted into a tomographic dataset. For this purpose, the open source tools *IMOD* and *FIJI* are used, which process the image data with the help of a filtered back-projection algorithm and adapted macros [14,15]. The resulting tomographic images can finally be displayed with any volumetric imaging program.

### 2.2. SLOT laser parameter adjustment and manual sample alignment

In this paper, two SLOT specific challenges will be addressed. To understand the constraints for automated, alignment-free SLOT acquisition, it is necessary to first explain the conditions that must be met for optimal acquisition.

**Fig 2:**
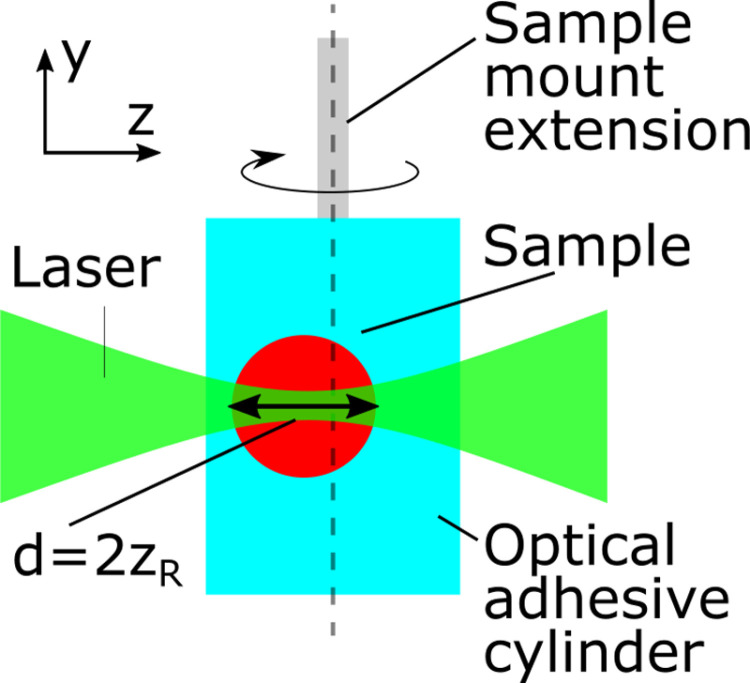
The dependency on the beam parameter and the sample size of a SLOT acquisition. The beam focus length is determined by the diameter of the sample, such that the focus length (represented by twice the Rayleigh length *z*_*R*_) must cover the sample size *d*.


The first important condition is the ratio of the laser beam focus length to the diameter of the sample to be examined, see Fig 2. The Rayleigh length of the laser beam should correspond to half the sample diameter [16].

$$\begin{array}{c} 2*z_{r}=d=2*\frac{n\text{*}\lambda }{NA^{2}} \end{array}$$ (1)

Where *z*_*R*_ represents the Rayleigh length, which must be equal to the half sample diameter *d*. The Rayleigh length can also be rewritten as a dependency of the refractive index *n*, the laser’s wavelength *λ* and the numerical aperture *NA* of the incident beam. This ratio gives the smallest possible beam focus (in diameter) still covering the entire sample lengthwise. This in turn ensures the highest possible optical resolution for each individual measurement. In SLOT, the Rayleigh length can be adjusted via the variable beam expander.

**Fig 3:**
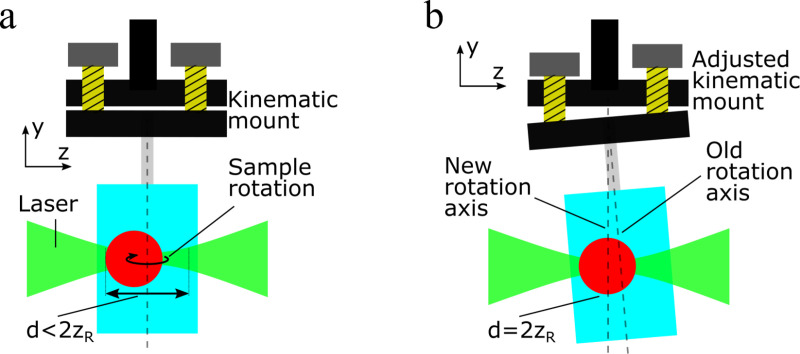
The manual sample alignment process. (a): The embedded sample is mounted on a kinematic mount. Due to a possible misalignment of the sample within the embedding material, the sample would wobble around the rotation axis. To gain a sharp image, the focus length must cover the entire wobble movement range of the sample, and meaning the focus length must be larger than the sample diameter. (b): After the alignment of the sample to the rotation axis of the mount, the focus length is shortened, compared to (a), to cover the sample’s diameter. This leads to a higher optical resolution.

This first condition leads to a second requirement which states that a sample should ideally rotate around its own center during a measurement, so that the sample diameter to be acquired remains as small as possible and does not vary. When the sample is not embedded within the middle of the polymer cylinder, a tumbling motion around the rotation axis is the result. The Rayleigh length of the beam will then have to cover the entire diameter of the rotational movement, which is considerably larger than the sample diameter. Since a longer focus of the Gaussian beam also results in a larger diameter, the resolution of the resulting images decreases. It is therefore necessary to manually re-align the entire cylinder, moving the sample back into the center of the acquisition space, i.e., the rotation axis of the rotation unit, see Fig 3. The sample, embedded in the adhesive, is mounted on a kinematic holder KC1L/M (*Thorlabs*, Germany) via an extension. The sample can then be aligned using a virtual rotation axis in the live image of the SLOT image output. The kinematic mount is adjusted via hand-eye-coordination.

Compliance with the conditions described above is crucial for the resulting image quality of the SLOT acquisition. Especially the manual alignment of the sample is strongly dependent on the experience of the user. This leads to the need to develop new methods to improve sample alignment for SLOT.

### 2.3. Field of view and focus tracking of a non-aligned SLOT sample

To ensure consistent image quality through repeatable measurements, as well as reduced sample preparation time through less manual user input, automation of this process must be developed. In this paper we present one of the possible methods that does not require a specific sample orientation.

#### 2.3.1. Sample tracking method overview.

**Fig 4:**
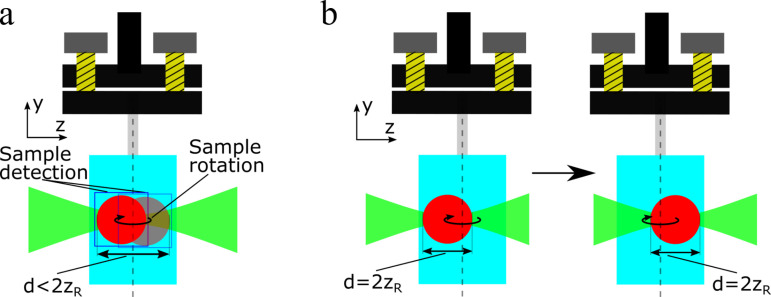
The principle of the new virtual alignment method. (a): The path of the non-aligned, wobbling sample is detected in a fast-pass SLOT acquisition with fewer angle projections. The images are processed with computer vision to calculate a movement prediction. (b): With the calculated movement of the sample, the focus can be shifted, so that it always covers the sample diameter, following the sample position.

The method developed here requires no additional hardware and few computer resources. Our computer vision-based approach uses existing imaging methods to calculate the movement of a misaligned sample during rotation. This information is then processed to adjust the parameters of the SLOT (laser parameters) and imaging. The focus tracking is performed as follows:

1) The sample is placed in the SLOT, with no alignment necessary. During a rotation, the sample may tumble around the axis of the rotation stage, depending on how it was embedded during the clearing process.
2) The parameters of the laser beam are adjusted with prefixed settings. Here, the focus length is set to fit the diameter of the glass cuvette (18 mm in diameter, resulting in an optical resolution of 34.01 µm). This ensures a sufficient imaging quality for the following object detection. The linear (focus) stage, which controls the position of the focus is aligned with the axis of the rotation stage.
3) To determine the sample movement, the sample is rotated in a fast pass prior to the actual acquisition, meaning that the rotation stage is not stopped for image acquisition and fewer projection angles are covered. During the rotation, images are taken with full Field of View (FoV), utilizing the entire scan area of the SLOT. This ensures that the sample cannot move out of the scan area and the sample can be detected during the full rotation. The rotation speed is set lower than the usual speed and the images are acquired with 2 seconds integration time. This results in a total scan time of 52 seconds, where 26 images are acquired.
4) The acquired images of the fast pass are subsequently passed to the image processing algorithm, which is describe in detail later. Here the sample is detected and its position is calculated for each passed image, see Fig 4a. The information is used to calculate the sample’s movement during the rotation.
5) After data processing, the image acquisition parameters can now be adjusted to the actual acquisition. The focus length of the laser is adjusted to fit the sample diameter, ensuring the optimal optical resolution. The FoV is set to the desired area, ensuring that only relevant data is acquired and the minimum acquisition time is provided.
6) Finally, the acquisition process is started. According to the calculated movement, the focus stage moves to its initial position, where the focus of the laser is aligned with the sample’s diameter. During the rotation of the sample, for each discrete projection angle, the focus stage is moved, see Fig 4b. This way, the FoV is shifted to follow the sample, so that it always stays in the middle of the FoV. Before each projection acquisition the system waits for the stages movements in order to avoid motion artifacts caused by their movement.

#### 2.3.2. Sample detection and movement calculation algorithm.

**Fig 5:**
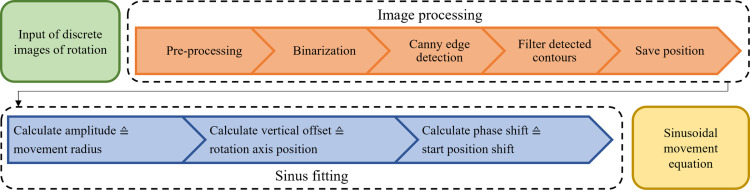
The process chain of the sample movement calculation. First, the images are processed with a set of computer vision algorithms to detect the sample. Next, the sample positions are calculated and fitted into a sinusoidal movement equation. This equation finally represents the movement of the sample during its non-aligned rotation.

To calculate the movement of a non-aligned sample (point 4) of chapter 2.3.1), we created an algorithm which detects the samples position during the fast-pass rotation and finally calculates a sinusoidal movement equation that represents the sample movement. Fig 5 shows the process chain.

First, the acquired images are passed to the image processing chain. Here we utilize the open source image processing library *OpenCV* [17]. The input images are pre-processed with a set of standard functions (histogram equalization, unsharp mask, median blurring and morphological opening). Next, we apply a binarization with an Otsu-threshold. To detect the sample, a Canny edge detection is applied to the binarized images. To calculate the sample position, we filter the detected contours to exclude predictable interference factors, such as the outlines of the glass cuvette or unwanted dust particles or gas bubbles which haven’t been excluded by the pre-processing chain. Subsequently, a bounding box enclosing the samples contours is calculated. The center of the bounding box represents the sample coordinate, which are saved for each acquired and processed image.

Secondly, the sinusoidal movement of the sample in image coordinates is calculated with the primary calculated sample positions, according to the following equation:


$$\begin{array}{c} S=a*\sin\left(\varphi +c\right)+d \end{array}$$ (2)

Here, *S* represents the center of the sample in pixel coordinates in y-direction. *a* is the radius if the tumbling movement, *φ* represents the current angle position of the rotation stage and subsequently the sample, *c* is the phase shift of the initial sample position and *d* the vertical shift of the rotation axis in pixel coordinates. This formula describes the movement of the adapted FoV, which is set to the beginning of a usual SLOT acquisition. This ensures that the sample does not move out of the FoV if the boundaries are set tight to the sample. A second sinusoidal equation, describing the movement of the focus is given by:


$$\begin{array}{c} F=a*\cos\left(\varphi +c\right)+stage\_position_{initial}-a*sin\left(c\right) \end{array}$$ (3)

Here, *F* represents the position of the focus in z-direction, regarding the focus stage. *a*, *φ* and *c* are corresponding values to Equation (2). The variable *stage_position*_*initial*_ represents the initial position of the focus at the beginning of the SLOT acquisition at the initial angle. The last part of the equation subtracts the part caused by the rotation of the corresponding coordinate direction.

The two described equations can now be used to control the adjustment of the field of view and the position of the focus, ensuring that the image is acquired with high quality by narrowing the focal length to the shortest possible length, resulting in the highest possible optical resolution.

### 2.4. Jitter correction for transparent rotating samples

After data acquisition, the sample may tremble during rotation. This can be observed regardless of whether manual alignment or automated focus tracking was performed. This remaining movement error is particularly noticeable with relatively small samples, such as cell spheroids or organoids. This is caused by mechanical irregularities in the rotation stage during the rotation, which then affect the movement of the sample. When reconstructing this data, this leads to artefacts in the reconstructed images in the form of blurred edges or double walls. So far, an algorithm based on Birk et al. [18] has often been used in SLOT to correct this error. This method corrects the sinogram of the data set using a sine fitting of prominent points in the image. When using homogeneously transparent samples however, one encounters the problem that no distinctive points can be found. We have therefore developed a new algorithm that is able to detect samples regardless of their composition and corrects the tremor movement.

#### 2.4.1. Sample detection and jitter correction algorithm.

For our new automated approach, we developed an algorithm based on Wang et al. [19], which utilizes the geometric moments of computationally detected objects. We have developed a Python-based user interface for this purpose (utilizing the *scikit-image* and *scipy* library [20,21]), with the help of which the image data sets can be processed in a user-friendly way.

**Fig 6:**
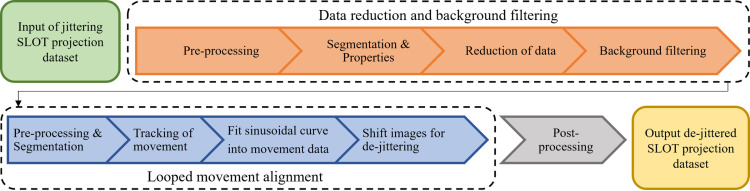
The process chain of the de-jitter algorithm. First the sample is detected and segmented. Next the object properties are extracted, whereas the geometric centroid is stored as the object’s movement coordinate. The tracked coordinates are then fitted to a sinusoidal curvature. The images are finally shifted in order to fit to the sinusoidal curve.

The first part reduces the amount of processed data and filters the background. This helps the further processing to identify the sample more easily. The captured raw image data is first loaded into the program. The program then takes over the complete processing of the image data, see Fig 6. The first step is pre-processing, including background-subtraction, filtering, binarization and morphological operands. The background subtraction is optionally done with the rolling-ball algorithm, originated from Sternberg et al. [22]. A gaussian blur is used for filtering, reducing artifacts of small particles as a result. The binarization is performed with a gaussian mixture model, whereby an Otsu threshold or a canny edge detection including surface filling can also be selected. Finally, optional morphological opening is also available which reduces imaging artifacts, such as dust particles. After pre-processing, the images are labelled using a region-growing based segmentation process. The labelled images are analyzed and the object to be detected is isolated using the segmentation parameters. The information of the segmentation is then used to reduce the amount of data, by reducing the unnecessary image part above and underneath the detected object. The background of the image is removed as well to ease the object detection in the further steps.

The second part of the algorithm tracks the object and corrects the jitter artifacts. This entire part is done repeatedly until the jitter error is neglectably small. Within this loop, first, the reduced, background removed images are pre-processed again with the same set of functions as before, as well as the segmentation steps to detect the object. In order to reduce the segmented object to a traceable point, the geodesic center is determined according to Freeman et al. [23], with the implementation of *scikit-image*’s centroids. This allows the center of gravity of a non-symmetrical object to be tracked. Alternatively, the center point of the enclosing bounding-box can also be used here, but this is only suitable for symmetrical objects in order to save computational resources. A sine curve is fitted to the saved tracked centroid points. This curve represents the course of the sample’s axis of rotation within the SLOT image. The difference between the original image and the corrected file is then calculated for each image and the original image is shifted horizontally by the corresponding number of pixels. This results in a uniform rotational movement of the sample in the data set. Instead of a sine fit, it is also possible to fit the sample to the middle of the image, which results in the sample turning around its own axis and a reduced dataset size. Lastly, these steps are repeated, whereas a resulting jitter-error is calculated, which has to be reduced until an abortion criterion is met. We also included an optional vertical shift. This step is important to samples with a large displacement. These displacements may induce a vertical shift to the sample if the rotation axis is angled, especially visible in small samples.

Finally, the images can be post-processed using SLOT-specific image processing methods, such as reducing the image size in vertical and horizontal direction or background removal.

### 2.5. Validation of the correction algorithms with cleared spheroids

**Fig 7:**
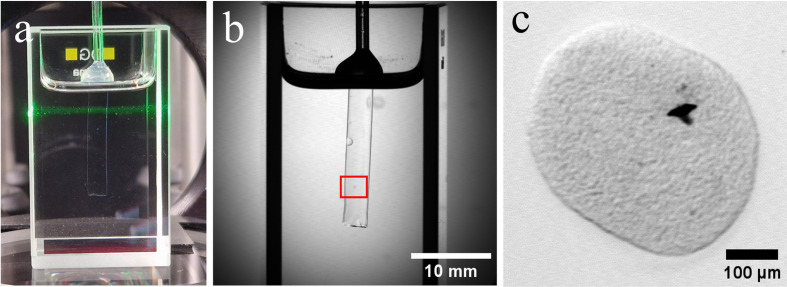
The embedded cell spheroid, used as a test target. (a): Image of the embedded sample in the SLOT during acquisition. The sample is embedded in a polymer cylinder and placed in a cuvette filled with refractive index matched silicone oil. The green laser scans across the sample. (b): The full field of view image acquired with the transmission channel of SLOT. (c): The used cell spheroid, consisting of 100.000 human osteoblast cells. The sample is located in the red square of (b).

To validate our newly developed algorithms, we created an application-oriented test sample. In order to test the possibilities of the developed algorithm, it is essential that the sample used fulfills several conditions. From the perspective of algorithmic testing, the sample should be a challenge to the algorithm in order to identify potential weaknesses. Secondly, the same sample should be processed by both algorithms in order to examine possible interactions of the corrections. Ultimately, the sample should be examined with SLOT under real conditions. For these reasons, cell spheroids were selected. These 3D cell constructs represent the smallest objects that have been examined with SLOT to date. They are difficult to detect and susceptible to jitter. In addition, cleared spheroids are usually homogeneous and do not provide any clues for regular jitter correction options through clearly identifiable points within their structures. Here, we used a cell spheroid, consisting of approximately 100.000 human osteoblast cells, see Fig 7, provided by *InSCREENeX* (Germany). The sample was cleared with the CRISTAL protocol, where benzyl alcohol (*Carl Roth*, Germany) was used for the clearing solution and NOA 68 (*Norland Optical Adhesive*, USA) for the embedding material. The embedded sample was submerged in a cuvette, filled with silicone oil. The oil consists of a mixture of *AP150Wacker* (*Sigma-Aldrich*, Germany) and *Tetra* (*Indomet*, Germany) with a resulting refractive index of *n* =  1.54, which matches both the NOA 68 and the cleared sample.

The prepared sample was acquired with a basic SLOT setup, utilizing a 532 nm laser diode. Since we decided on a relatively small and transparent (cleared) sample, we could not use the full field of view for the automated sample detection. For this reason, the user needed to roughly select an appropriate image area in which the sample moves during the focus tracking. This entry could be made freely by the user and only served to initially limit the detectable area. The ratio of the resulting zoom into the field of view is taken into account accordingly when calculating the circular path. Tracking the sample in the transmission channel was still error prone due to its high transparency. Therefore, the fluorescence channel was used for detection. For this purpose, the autofluorescence of the sample was detected using a photomultiplier tube and an 578/16 fluorescence filter (*Edmund Optics*, Germany). The resulting image was then inverted in the pre-processing step and subsequently processed using the algorithm described above.

## 3. Results

In this paper we present two approaches with the respective aim of increasing the degree of automation and improving the quality of SLOT recordings using automated methods. To validate our newly developed algorithms, we created a test sample and used both methods on the same sample.

### 3.1.Field of view and focus tracking results

**Fig 8:**
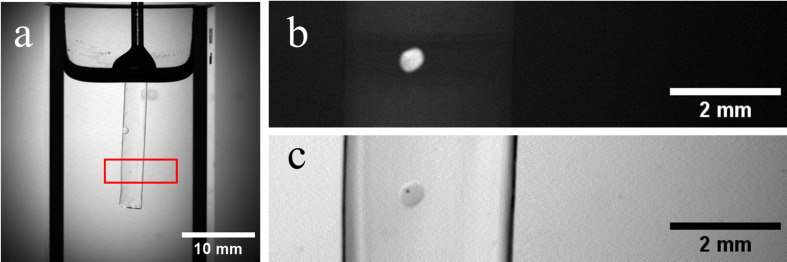
The setup for the sample tracking algorithm. (a): An overview of the field of view used in the tracking algorithm. The red rectangle shows the zoomed field of view that was necessary due to the small sample size. (b): The autofluorescence signal in the photo multiplier channel of the zoomed field of view. The cell spheroid emits a detectable signal between 570 and 586 nm. (c): The transmission signal from the photodiode of the zoomed field of view. The spheroid traveled 1920 µm in diameter around the rotation axis.

In the first algorithm, sample tracking for SLOT was implemented, which adjusts both the FoV and the focus position to match the sample position during a SLOT measurement. For this purpose, the sample presented in section 2.5 was analyzed in the SLOT. Fig 8 shows the image section used for the analysis. Due to the small sample size and its high transparency, we were unable to use the entire FoV as there were too many interfering factors affecting sample recognition. Even small impurities within the sample or tiny air inclusions were sufficient to disrupt the algorithm. The solution consisted of limiting the FOV to a rough alignment placed by the user, see Fig 8 (a). Further, the fluorescence channel of the SLOT was used for sample detection, as the sample can be clearly identified in the fluorescence image, see Fig 8 (b). The focus length was set to the maximum length during the determination of the circular path. As a result, the sample was imaged with the same focus during the entire rotation. A video of the sample movement can be found in supplement video S1.

**Fig 9:**
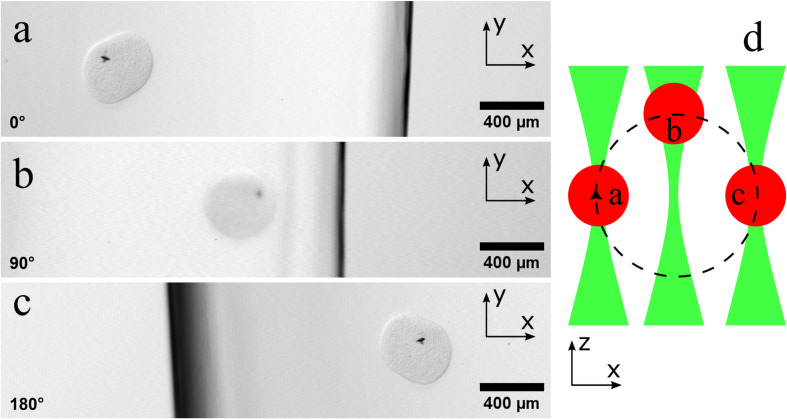
Movement of the sample during the rotation with a fixed focus position. (a): The sample is in focus. The focus length is set to the sample diameter. The image shows the sample on the farthest left position. (b): After a 90-degree rotation, the sample has moved the furthest from the initial focus point and is in the center of the image. The sample appears blurry because it is no longer in focus. (c): After a total of 180-degree rotation, the sample is in focus again and is now on the opposite side of the rotation axis from the initial position in (a). (d): The principle of the movement of the sample and the focus position of the laser is shown in the schematic drawing.

To better understand what the movement and the shift in focus mean, Fig 9 shows the movement of the used sample during a rotation where the focus was set to the sample size but the focus position was not moved with the sample. In Fig 9 (a), the sample is at the maximum deflection on the left side of the image. Here the sample is in focus and is therefore displayed sharply. After a rotation of 90 degrees, the sample is in the middle of the rotation axis in the image section, see Fig 9 (b). Here the sample is furthest away from the fixed focus point, which leads to a blurred image. After a further rotation of 90 degrees, the sample is back in focus and on the opposite side of the rotation axis, see Fig 9 (c). Here the sample is in focus again because it is congruent with the focus. In our experiment, the sample rotated on a radius of 1920 µm around the axis of rotation of the rotation stage during the recording.

**Fig 10:**
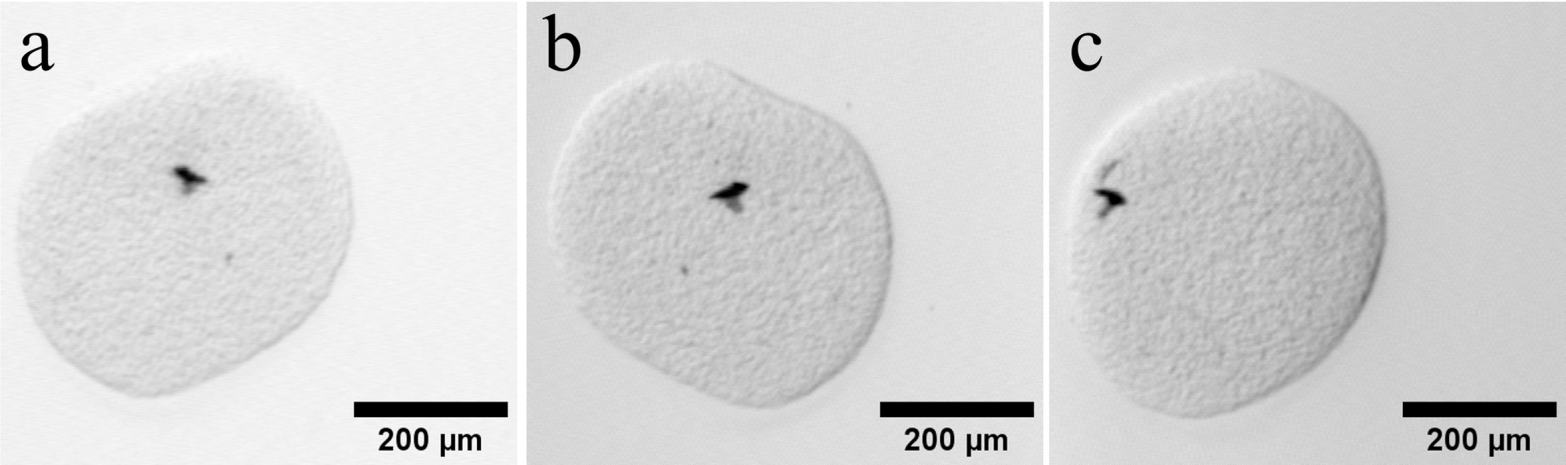
Excerpts from the raw acquired data set with the tracking algorithm. (a)-(c): The spheroid shown in different angles. The image retains its sharpness in all angles, which means that the focal length of the laser was successfully shifted with the movement of the sample. The sample position has an offset to the left side of the image in all positions. The background of the data differs from angle to angle, caused by the path of the light through the sample embedding.

After the determination of the circular motion of the sample, as described earlier, an acquisition was started with the activated movement of the FoV and the focus. First, a FoV was selected like for a normal measurement, in which only the sample is visible in the image. Secondly, the focus length of the laser beam was set to correspond to the sample diameter and the focal plane was set to the position where the sample is imaged most sharply. The result of the acquisition can be seen in Fig 10 and in the supplemental material S2.

The images of Fig 10 (a-c) show excerpts of the unprocessed raw dataset. The sample retains its sharpness in all angles, meaning that the narrow focal length and its focus point of the laser beam was successfully shifted alongside with the movement of the sample. Otherwise, the image would have been blurred as shown in Fig 9 (b). Furthermore, the acquired dataset shows an offset of the sample towards the left of the image. The FoV had been selected to place the sample in the center of the image. The cause of the offset that occurred here is still being investigated. Nevertheless, the sample did not move out of the image during rotation and only exhibited a very small circular motion, which will be discussed in more detail in the next section 3.2. This residual movement may have resulted from the small sample size, since even small measurement deviations of a few pixels can have a comparatively large effect here. The change of the brightness in the images is caused by the embedding material. The sample position is not on the middle axis of the cylindrical embedding material. At the same time, the slightest deviations in the refractive index matching between the embedding material and the silicone oil weakly refract the incident light of the laser beam. Depending on the position of the sample and the corresponding angle of incidence, this results in a different brightness of the image.

### 3.2. Dejitter of the acquired datasets

With the second algorithm, a method was implemented to dejitter the acquired data from movement artifacts. To test the developed algorithm, we used the unprocessed data from section 3.1. The algorithm was developed to correct jittering movements even of homogeneous structures. However, the cell spheroid used here also contained inhomogeneous structures, which can be seen as dark spots in Fig 7 (c). These are most likely cavities of dead cells, which cause dark discoloration due to their higher degree of absorption. This circumstance allows us to investigate the sinograms of the acquired and the processed dataset with a distinctive structure to analyze.

**Fig 11:**
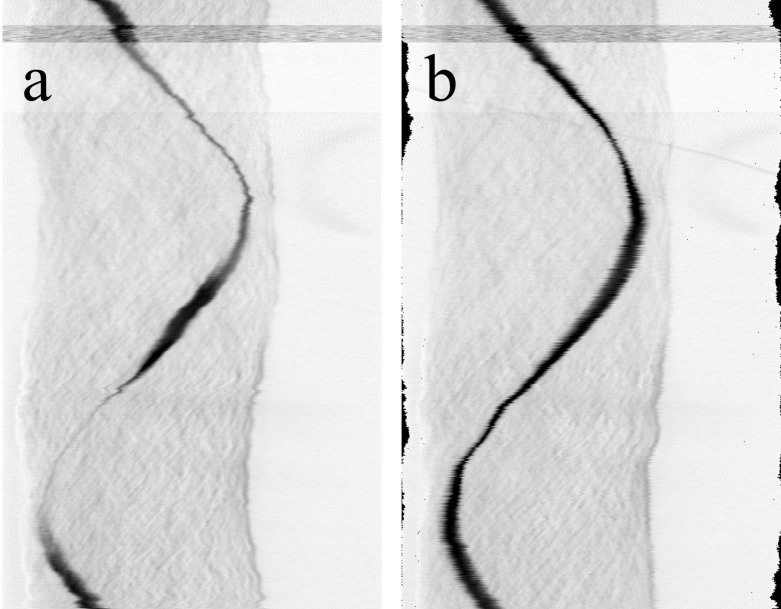
A sinogram of the test sample before and after the dejitter algorithm. The black sinusoidal line represents the dark spot near the z-center of the sample seen in the transmission projections (Figs 7-10). (a): The uncorrected raw data, acquired with the tracking algorithm. The sinusoidal lines appear jittery due to the irregular wobble movements of the rotation stage. (b): The dejittered dataset. The sinusoidal lines appear smoother and have a constant intensity. The size of the x-shift per line can be seen at the edge of the image as dark pixels.

When applying the new algorithm, a rolling-ball background subtraction, a Gaussian filter and Otsu’s thresholding were first carried out for pre-processing. The tracking method used was the centroids method. The processing time for the data set, consisting of 800 images of 500x477 pixels, was recorded in several runs with an average processing time of 105.69 seconds for the settings described in here. In Fig 11 a sinogram of the dataset before and after the processing can be observed and is also observable in the supplemental material S3. We chose the sinogram at the height of the dark spot near the z-center of the sample shown in Figs 7-10.

In this Fig 11 (a), one of the unprocessed sinograms of the acquired dataset can be seen. Here, several artifacts are present. The jitter artifact on which we focus in this work is illustrated here by the black sinusoidal envelope. Each deviation from the sinusoidal curve represents an irregular movement of the sample from its circular motion. Here, deviations from the sinusoidal curve can be found throughout in various degrees of intensity. Another disturbing artifact for an optimal reconstruction is the vertical movement of the sample in the dataset, which is represented here by the loss of intensity in the lower third of the image.

The corrected dataset after the application of the dejitter algorithm can be seen in Fig 11 (b). Here, the dark sinusoidal line has been smoothed and appears more constant. The amount of shifted pixels per line can be seen at the edges of the image as black spots. There still remains a jitter in horizontal direction, noticeable at the edges of the sinusoidal lines that display an x-shift of a few pixels in irregular patterns. This is a residual artifact of the jitter correction which was neglected in this work but is subject to further investigations. The vertical shift induced in this sample is an artifact caused by an initially tilted rotation axis in relation to the angle of the incident laser beam. This artifact was mechanically corrected, but due the relatively small size of the sample used in here, there still remained a small vertical movement which was corrected by our algorithm as well. The vertical movement correction is visible here as a more constant intensity along the sinusoidal line, compared to Fig 11 (a).

### 3.3. Reconstruction of the corrected dataset

**Fig 12:**
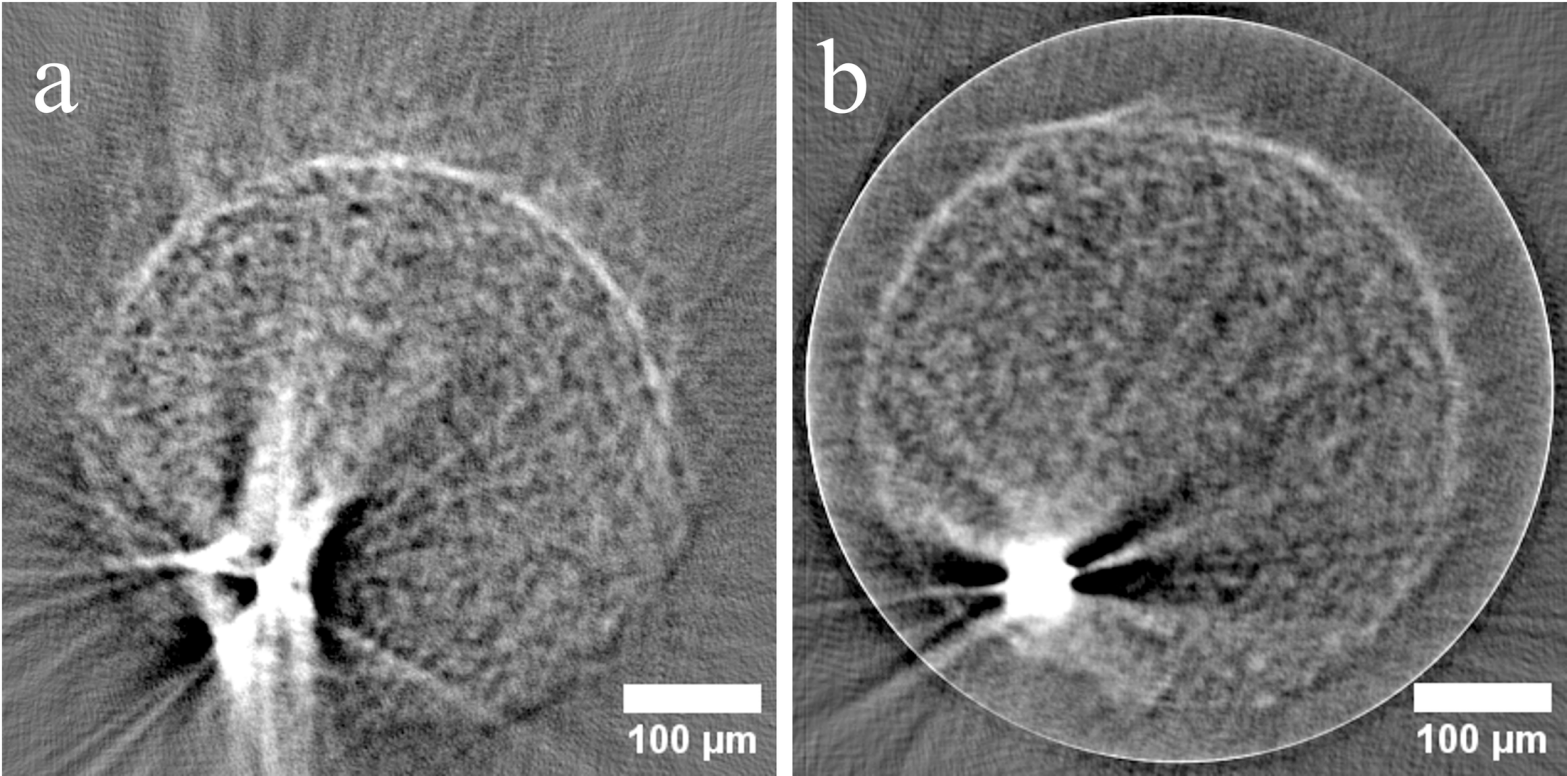
The reconstruction of the sinogram of the same image slice as shown in Fig 11. The slice was reconstructed with the filtered back-projection algorithm. (a): The tomogram of the uncorrected dataset. The structures are blurred and show unsharp edges and doubling artifacts. (b) The tomogram of the jitter corrected dataset. Here, the edges of the sample are sharper and the reconstruction artifacts are reduced. The quality of the image has improved.

To evaluate the resulting effect of our algorithms, we reconstructed the datasets of the uncorrected and of the dejitter-corrected datasets. We used the same image as for Fig 11. The filtered back-projection algorithm of *IMOD* was used for reconstruction. The result can be seen in Fig 12.

In Fig 12 (a) the tomogram of the uncorrected dataset is shown. Here, the round shape of the spheroid is recognizable. However, this round structure appears to be doubled. This double appearance is a result of the unintentional movement of the sample during the rotation, as well as the movement in the vertical direction due to the above-described residual tilt of the axis. Another artifact can be seen in the bright structure at the bottom left of the image. Due to the motion artifacts, the dark cavity is interspersed with stripe artifacts. These are typical reconstruction artifacts that arise from the jittering motion.

The reconstructed jitter-corrected dataset can be seen in Fig 12 (b). Compared to the uncorrected reconstruction, the structures appear sharper. The double contours of the sample are no longer present. The area of the reconstruction of dark cavity in the lower left of the image is significantly reduced. The stripe artifacts are also significantly reduced here. These are all indications that the dejitter algorithm has successfully corrected the motion artifacts. The outer ring in the image is a typical artifact of the reconstruction algorithm and can be neglected as it does not overlap with the sample reconstruction.

## 4. Discussion

In this work, we developed and tested two algorithms to automate sample alignment and correction of motion artifacts for SLOT acquisitions. The improvements of the original SLOT method [4] thus result in an increase in image quality, as well as the guaranteed reproducibility of measurement results.

The first algorithm was developed to eliminate the need for manual alignment of the sample. For this purpose, a computer-aided sample tracking system was developed that analyzes the movement of the sample, forwards the calculated movement profile to the acquisition program and ultimately ensures that the sample remains in the image field of view during the acquisition and is in focus at all times by shifting the focus of the imaging laser beam. As an alternative to the solution approach presented here, it would also be possible to automate the physical alignment of the sample. This could be achieved by extending the kinematic sample holder with the help of electric motors. The motorization in combination with computer-aided sample detection would map the alignment process analogous to manual alignment. This would however require expensive precision motors to produce a similar result. The experiments carried out here confirmed the functionality of the algorithm. A cell spheroid was chosen for validation which represents a challenging sample, as it was both very small and highly transparent, making it difficult to detect. This challenge was met by using the ability of SLOT to take fluorescence images simultaneously, which facilitated sample detection. Some user interference was required at this point to pre-select the rough area where the sample was located to prevent reflection artifacts from interfering with sample detection. The sample detection was implemented here using classical methods. For consistent samples, artificial intelligence-based detection methods can be used in the future, which can improve sample detection for specific samples, as is already possible for spheroid-like organoids [24,25]. A conspicuous offset of the sample in relation to the image center was observed after focus alignment (Fig 10), the origin of which is still unknown. In our experiments, the sample did not move out of the image area, but this should be analyzed and corrected in the future. Finally, the initial alignment of the axes of the kinematic mount turned out to be an influencing factor for the acquisition. A small inaccuracy here, especially for very small samples like the cell spheroids, can cause a small movement of the sample in the vertical direction. This is not a challenge for sample tracking, but it does lead to artifacts in the reconstruction in general.

The second algorithm was developed to remove motion artifacts from images of samples in the SLOT. For this purpose, an algorithm was developed that is able to correct even homogeneous samples. The challenge here was to develop an algorithm that does not only work for a specific sample type or a sample featuring a distinctive detail [18], as SLOT is used to analyze a wide variety of samples, among them homogeneously cleared samples. With our test sample, we were also able to validate the functionality of the second algorithm. This showed that the positioning of the sample within the embedding material can be decisive. If the sample is too close to the edge, this leads to irregular light conditions in the background of the sample. If these are too strong, the program can no longer segment the sample correctly, which is essential to its functionality. To further boost the imaging quality, it should be ensured that the samples are located in the middle of the embedding material or that alternative embedding concepts, such as agarose [26] or hydrogel [27] embedding, are used that can facilitate the handling of sample preparation. Our results have shown that the movement artifacts of the sample have been significantly reduced, which has led to a significant improvement in image quality. However, there remains a small residual movement of the sample of one pixel in x- and y-direction. This can be seen particularly in the sinogram on the dark sinusoidal structure, as it appears slightly blurred at the edges instead of having a clear edge. This residual artifact can be improved by integrating a smoothing algorithm for motion correction.

Regarding computational resources we observed that no high hardware requirements are necessary. The first algorithm was performed directly on the acquisition computer. Here, the most resource-intensive processes were the image processing steps. These were carried out in a few milliseconds on our mid-range system and thus had no significant impact on the system’s performance. The second algorithm required more processing time between 100 and 200 seconds. This was caused by the code itself that is not yet optimized for parallel processing or graphic processing unit usage. With such optimization the computational time could be highly decreased in the future.

## 5. Conclusion

Two algorithms were developed in this work for the automation of SLOT imaging. The first algorithm provides automated sample detection, which eliminates the need for manual, time-consuming and user-dependent sample alignment. This leads to a higher reliability of the system, as the repeatability is increased and the quality of the images, explicitly the effective optical resolution, is improved. The functionality of the sample tracking system was validated using a cell spheroid as a test sample. This could be reliably tracked and recorded. Despite the tumbling motion, the spheroid remained within a very limited image section. The beam focus was also successfully shifted so that the sample was always in focus. The second algorithm developed in this work is designed to reduce unwanted motion artifacts caused by sample jitter in the recorded SLOT dataset. The approach pursued here consists of detecting and segmenting the sample in the data set. A subsequent motion analysis and correction using image processing methods ensures a reduction of the interfering artifacts. A reduction of the movement in both the primary horizontal and secondary vertical direction was achieved. The correction of the sample movement led to a significant improvement in the reconstruction of the tomographic images.

Overall, it was possible to show that the algorithms developed not only work well individually, but above all that they work very well together.

## Supporting information

S1 File Supplemental video S1 showing the movement of the sample in a wide FoV. (MP4)

S2 File Supplemental S2 showing the result of the sample and focus tracking algorithm of the acquired image stack. (MP4)

S3 File Supplemental S3 showing the result of the dejittered image stack. (MP4)

## References

[1] Sharpe J, Ahlgren U, Perry P, Hill B, Ross A, Hecksher-Sørensen J, et al. Optical projection tomography as a tool for 3D microscopy and gene expression studies. Science. 2002;296(5567):541–5. doi: 10.1126/science.1068206 11964482

[2] Fujimoto JG. Optical coherence tomography: technology and applications. IEEE/LEOS International Conference on Optical MEMs. 2002.147–8. doi: 10.1109/omems.2002.1031485

[3] Stelzer EHK, Strobl F, Chang B-J, Preusser F, Preibisch S, McDole K, et al. Light sheet fluorescence microscopy. Nat Rev Methods Primers. 2021;1(1):. doi: 10.1038/s43586-021-00069-4

[4] Lorbeer R-A, Heidrich M, Lorbeer C, Ramírez Ojeda DF, Bicker G, Meyer H, et al. Highly efficient 3D fluorescence microscopy with a scanning laser optical tomograph. Opt Express. 2011;19(6):5419–30. doi: 10.1364/OE.19.005419 21445181

[5] Nolte L, Tinne N, Schulze J, Heinemann D, Antonopoulos GC, Meyer H, et al. Scanning laser optical tomography for in toto imaging of the murine cochlea. PLoS One. 2017;12(4):e0175431. doi: 10.1371/journal.pone.0175431 28388662 PMC5384786

[6] Kellner M, Heidrich M, Beigel R, Lorbeer R-A, Knudsen L, Ripken T, et al. Imaging of the mouse lung with scanning laser optical tomography (SLOT). J Appl Physiol (1985). 2012;113(6):975–83. doi: 10.1152/japplphysiol.00026.2012 22797312

[7] Kamin H, Nolte L, Maurer J, Stickeler E, Heinemann D, Heisterkamp A, et al. Correlative imaging and quantification of the tumor microenvironment of triple-negative-breast-cancer using lightsheet microscopy, scanning laser optical tomography, and TPEF (Conference Presentation). Multimodal Biomedical Imaging XVIII. 2023;4. doi: 10.1117/12.2648373

[8] Hohenhoff G, Meyer H, Jaeschke P, Ripken T, Kracht D, Kaierle S. Comparison of SLOT and μ-CT investigation of 3D printed polymer parts for quality assurance. Journal of Laser Applications. 2020;32(2):. doi: 10.2351/7.0000084

[9] Heidrich M, Kühnel MP, Kellner M, Lorbeer R-A, Lange T, Winkel A, et al. 3D imaging of biofilms on implants by detection of scattered light with a scanning laser optical tomograph. Biomed Opt Express. 2011;2(11):2982–94. doi: 10.1364/BOE.2.002982 22076261 PMC3207369

[10] Nolte L, Antonopoulos GC, Rämisch L, Heisterkamp A, Ripken T, Meyer H. Enabling second harmonic generation as a contrast mechanism for optical projection tomography (OPT) and scanning laser optical tomography (SLOT). Biomed Opt Express. 2018;9(6):2627–39. doi: 10.1364/BOE.9.002627 30258678 PMC6154203

[11] Kellner M, Heidrich M, Lorbeer R-A, Antonopoulos GC, Knudsen L, Wrede C, et al. A combined method for correlative 3D imaging of biological samples from macro to nano scale. Sci Rep. 2016;6 35606. doi: 10.1038/srep35606 27759114 PMC5069670

[12] Benecke H, Johannsmeier S, May T, Ripken T. Investigation of a hyperspectral Scanning Laser Optical Tomography setup for label-free cell identification. Sci Rep. 2024;14(1):17861. doi: 10.1038/s41598-024-68685-0 39090238 PMC11294569

[13] Heidrich M. SLOT: Eine auf optischer Computertomographie basierende Bildgebungsmethode für die Lebenswissenschaften. Gottfried Wilhelm Leibniz Universität Hannover. 2015.

[14] Kremer JR, Mastronarde DN, McIntosh JR. Computer visualization of three-dimensional image data using IMOD. J Struct Biol. 1996;116(1):71–6. doi: 10.1006/jsbi.1996.0013 8742726

[15] Schindelin J, Arganda-Carreras I, Frise E, Kaynig V, Longair M, Pietzsch T, et al. Fiji: an open-source platform for biological-image analysis. Nat Methods. 2012;9(7):676–82. doi: 10.1038/nmeth.2019 22743772 PMC3855844

[16] Kubitscheck U. Fluorescence Microscopy: From Principles to Biological Applications. Kubitscheck U, editor. Fluorescence Microscopy: From Principles to Biological Applications. Wiley; 2013. doi: 10.1002/9783527671595

[17] Gary B. The OpenCV Library. Dr Dobb’s Journal of Software Tools. 2015;25: 120–123.

[18] Birk UJ, Rieckher M, Konstantinides N, Darrell A, Sarasa-Renedo A, Meyer H, et al. Correction for specimen movement and rotation errors for in-vivo Optical Projection Tomography. Biomed Opt Express. 2010;1(1):87–96. doi: 10.1364/BOE.1.000087 21258448 PMC3005161

[19] Wang S, Liu J, Li Y, Chen J, Guan Y, Zhu L. Jitter correction for transmission X-ray microscopy via measurement of geometric moments. J Synchrotron Radiat. 2019;26(Pt 5):1808–14. doi: 10.1107/S1600577519008865 31490173

[20] Van Der Walt S, Schönberger JL, Nunez-Iglesias J, Boulogne F, Warner JD, Yager N, et al. Scikit-image: Image processing in python. PeerJ. 2014;2014: e453. doi: 10.7717/PEERJ.453/FIG-5 25024921 PMC4081273

[21] Virtanen P, Gommers R, Oliphant TE, Haberland M, Reddy T, Cournapeau D, et al. SciPy 1.0: fundamental algorithms for scientific computing in Python. Nat Methods. 2020;17(3):261–72. doi: 10.1038/s41592-019-0686-2 32015543 PMC7056644

[22] Sternberg SR. Biomedical Image Processing. Computer. 1983;16(1):22–34. doi: 10.1109/mc.1983.1654163

[23] Freeman LC. Centrality in social networks conceptual clarification. Social Networks. 1978;1(3):215–39. doi: 10.1016/0378-8733(78)90021-7

[24] Maramraju S, Kowalczewski A, Kaza A, Liu X, Singaraju JP, Albert MV, et al. AI-organoid integrated systems for biomedical studies and applications. Bioeng Transl Med. 2024;9(2):e10641. doi: 10.1002/btm2.10641 38435826 PMC10905559

[25] Du X, Chen Z, Li Q, Yang S, Jiang L, Yang Y, et al. Organoids revealed: morphological analysis of the profound next generation in-vitro model with artificial intelligence. Biodes Manuf. 2023;6(3):319–39. doi: 10.1007/s42242-022-00226-y 36713614 PMC9867835

[26] Azaripour A, Lagerweij T, Scharfbillig C, Jadczak AE, Willershausen B, Van Noorden CJF. A survey of clearing techniques for 3D imaging of tissues with special reference to connective tissue. Prog Histochem Cytochem. 2016;51(2):9–23. doi: 10.1016/j.proghi.2016.04.001 27142295

[27] Richardson DS, Lichtman JW. Clarifying Tissue Clearing. Cell. 2015;162(2):246–57. doi: 10.1016/j.cell.2015.06.067 26186186 PMC4537058

[28] Benecke H, Almadani F, Heske J, May T, Johannsmeier S, Ripken T. Automated focus tracking for non-aligned SLOT samples and tomographic jitter correction. Diffuse Optical Spectroscopy and Imaging IX. 2023;48. doi: 10.1117/12.2670894

